# Induced hepatic stellate cell integrin, α8β1, enhances cellular contractility and TGFβ activity in liver fibrosis

**DOI:** 10.1002/path.5618

**Published:** 2021-02-19

**Authors:** Norihisa Nishimichi, Kazuyuki Tsujino, Keishi Kanno, Kazuhiro Sentani, Tsuyoshi Kobayashi, Kazuaki Chayama, Dean Sheppard, Yasuyuki Yokosaki

**Affiliations:** ^1^ Integrin‐Matrix Biomedical Science Translational Research Center, Hiroshima University Hiroshima Japan; ^2^ Lung Biology Center, Department of Medicine Cardiovascular Research Institute, University of California San Francisco (UCSF) San Francisco CA USA; ^3^ General Internal Medicine Hiroshima University Hospital, Hiroshima University Hiroshima Japan; ^4^ Molecular Pathology Graduate School of Biomedical and Health Sciences, Hiroshima University Hiroshima Japan; ^5^ Gastroenterological and Transplant Surgery Graduate School of Biomedical and Health Sciences, Hiroshima University Hiroshima Japan; ^6^ Gastroenterology and Metabolism Graduate School of Biomedical and Health Sciences, Hiroshima University Hiroshima Japan

**Keywords:** integrin, fibrosis, αSMA, TGFβ, nephronectin, integrin α8β1, monoclonal antibody, drug, translation

## Abstract

No effective therapy exists for fatal fibrosis. New therapeutic targets are needed for hepatic fibrosis because the incidence keeps increasing. The activation and differentiation of fibroblasts into myofibroblasts that causes excessive matrix deposition is central to fibrosis. Here, we investigated whether (and which) integrin receptors for matrix proteins activate hepatic stellate cells (HSCs). First, integrin α‐subunits were investigated systematically for their expression over the course of HSC activation and their distribution on fibroblasts and other systemic primary cells. The most upregulated in plate culture‐activated HSCs and specifically expressed across fibroblast linages was the α8 subunit. An anti‐α8 neutralizing mAb was evaluated in three different murine fibrosis models: for cytotoxic (CCl_4_ treatment), non‐alcoholic steatohepatitis‐associated and cholestatic fibrosis. In all models, pathology and fibrosis markers (hydroxyproline and α‐smooth muscle actin) were improved following the mAb injection. We also CCl_4_‐treated mice with inducible *Itga8−/−*; these mice were protected from increased hydroxyproline levels. Furthermore, *ITGA8* was upregulated in specimens from 90 patients with liver fibrosis, indicating the relevance of our findings to liver fibrosis in people. Mechanistically, inhibition or ligand engagement of HSC α8 suppressed and enhanced myofibroblast differentiation, respectively, and HSC/fibroblast α8 activated latent TGFβ. Finally, integrin α8β1 potentially fulfils the growing need for anti‐fibrotic drugs and is an integrin not to be ignored. © 2021 The Authors. *The Journal of Pathology* published by John Wiley & Sons, Ltd. on behalf of The Pathological Society of Great Britain and Ireland.

## Introduction

The incidence of non‐alcoholic steatohepatitis (NASH) with fibrosis is growing, without any effective therapies. Hepatic stellate cell (HSC) activation and myofibroblast differentiation are central to hepatic fibrosis and occur upon deposition of extracellular matrix [1]. Integrins are major extracellular matrix receptors, so we reasoned that integrins induced on HSCs could play a critical role in this process. In the current study we systematically examined integrin α‐subunit expression over the course of activation of HSCs and found that the α8 subunit was most dramatically upregulated. α8 forms a heterodimer with β1 [2] and is expressed as α8β1 in smooth muscle cells and fibroblasts [3]. α8β1 is one of the eight integrins that recognize the tripeptide Arg‐Gly‐Asp (RGD), an integrin recognition motif that is present in the pro‐peptide of TGFβ1 and TGFβ3. Integrin binding to this motif is critical for integrin‐mediated TGFβ activation [4]. We therefore evaluated the functional significance of this integrin in multiple *in vivo* models and provide the mechanisms by which this integrin could modulate the behavior of activated HSCs.

## Materials and methods

HSCs were isolated from C57BL/6 mice and Wistar rats. Murine HSCs were used for expression studies and rat HSCs for the validation. Mechanistic studies employed rat HSCs to avoid possible allogenic effects from a mixture of murine HSCs. Sufficient α8 expression was confirmed before each experiment to avoid potential variability in the time course of α8 induction during activation of HSCs. Animal use and euthanasia protocols were reviewed and approved by the Animal Committees of Hiroshima University or University California San Francisco. For the use of human tissue, informed consent was provided by patients in accordance with the Declaration of Helsinki and approval from the Hiroshima University Institutional Review Board. Chicken neutralizing anti‐α8 recombinant mAb, YZ3, which reacts with most mammals, was generated in our laboratory [5] and characterized (see supplementary material, Figure S1). A chimeric form of YZ3 with mouse IgG_1_κ‐Fc was used throughout this study. All antibodies in this study are summarized in supplementary material, Table S1.

The initial global α8 knockout mouse line made in 1997 was characterized by kidney agenesis and perinatal death [6]. Contribution of α8β1 to nephrogenesis can be modified by ‘stochastic factors’, as the penetrance of the phenotype was only approximately 50% [6]. To reduce the possible compensation for loss of α8β1 function, we employed temporally inducible global deletion, *Itga8* ^flox/flox^;*Rosa26‐Cre* ^*ER*^, so that expression of α8β1 remains normal before induction of fibrosis. The shorter interval should avoid the effects of life‐long genetic compensation that may have affected results in previous studies in other organs [7, 8, 9].

Details of cells and culturing, antibodies, animals, RT‐qPCR, flow cytometry, experimental fibrosis, hydroxyproline assay, measurement of areas stained by Masson's trichrome or α‐smooth muscle actin (αSMA) immunostaining, human liver tissues, recombinant nephronectin proteins, western blotting, immunofluorescence, gel contraction assay, TGFβ activation assay and statistical analyses are provided in supplementary material, Supplementary materials and methods.

## Results

### α8 expression in HSCs and fibroblasts

**Figure 1:**
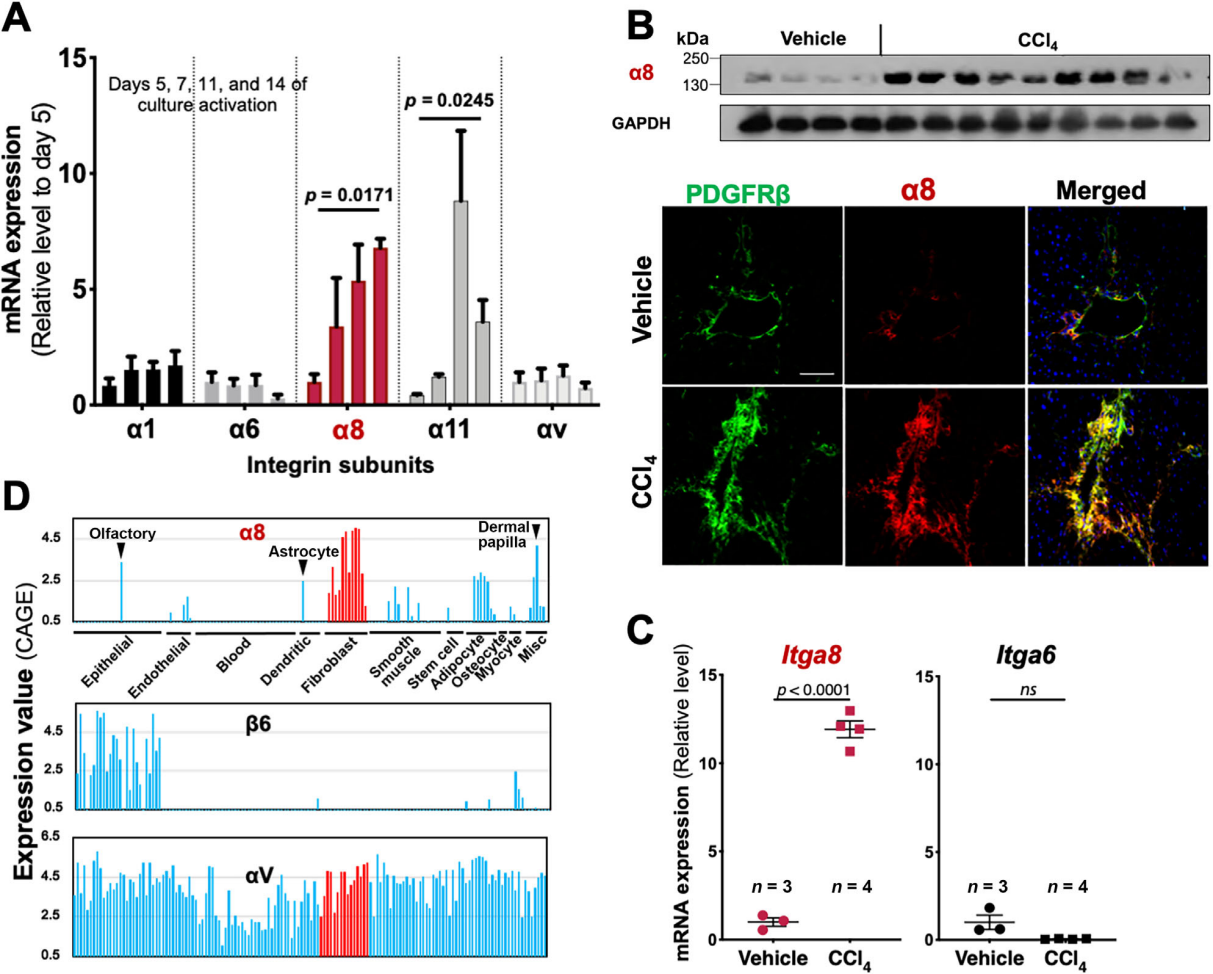
Induction and localization of integrin α8 subunit expression in HSCs/fibroblasts. (A) *Itga8* mRNA (RT‐qPCR) expression of murine HSCs in 14‐day culture. RT‐qPCR was repeated three times (*n* = 9 wells, pooled from three experiments). (B) α8 induction in CCl_4_‐treated liver by western blotting (top) and by immunofluorescence with α8 in red and PDGFRβ in green (bottom). (C) α8 induction in isolated *ex vivo* HSCs from CCl_4_‐treated liver by RT‐qPCR. (D) Expression of integrin subunit mRNAs for α8, β6, and αV in 144 primary cell lines. Data were retrieved from the FANTOM database and sorted by cell types indicated. Each bar represents a cell line.

Mouse HSCs grown on plastic for 14 days showed increased *Itga8* (Figure 1A; *p* = 0.0171) and *Itga11* mRNA expression. A similar increase in *Itga8* expression was observed in rat HSCs up to 27‐fold and four‐fold at the mRNA (see supplementary material, Figure S2A; 27‐fold, *p* < 0.0001) and the protein (see supplementary material, Figure S2B) level, respectively. Notably, α8 protein was not detected in quiescent HSCs at days 1 and 3. Integrin α6, which is constitutively expressed in HSCs [10], was used as a control. As culture‐activated HSCs mimic the fibrotic response [11], we explored α8 expression in HSCs in response to *in vivo* CCl_4_ treatment. Western blotting of whole liver lysate showed marked α8 upregulation. Immunofluorescence showed barely detectable α8 in normal livers and an increase in the periportal area colocalizing with PDGFRβ (Figure 1B). Furthermore, α8 was upregulated in HSCs isolated from CCl_4_‐treated mice (Figure 1C). Next, we analyzed α8 expression from databases analyzing fibroblasts and related linages. In 144 primary cells from various human tissues [12], *ITGA8* mRNA was expressed across 15 fibroblast lines but was less abundant in other cell types (Figure 1D), in contrast to integrin β6 or αv subunits. FACS analyses showed constitutive α8 expression in fibroblasts from the lung, heart, and kidney (see supplementary material, Figure S2C). Taken together, α8 was found to be expressed in HSCs/fibroblast linages, and was induced by activation of HSCs *in vitro* and *in vivo*.

### *In vivo* role of α8 in fibrosis

**Figure 2:**
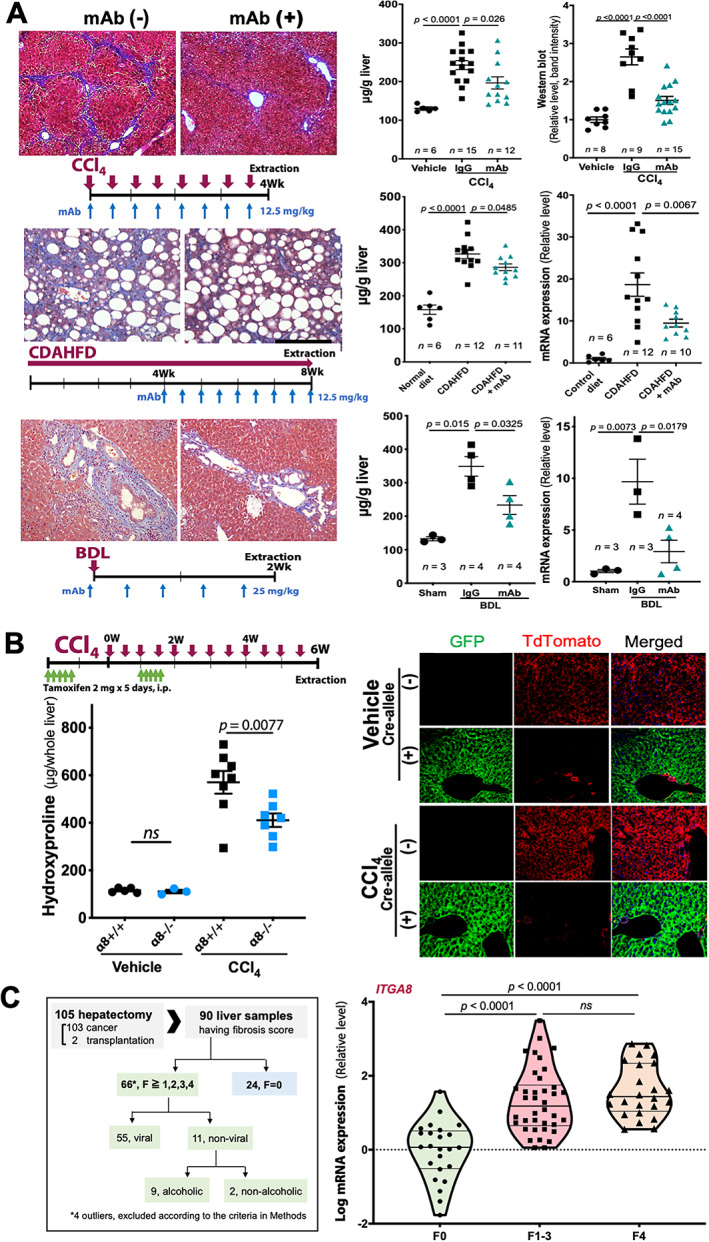
*In vivo* contribution of integrin α8β1 in mice and humans. (A) Effects of anti‐α8 mAb on liver fibrosis in three models: CCl_4_ (top), NASH (middle), and bile duct ligation (BDL; bottom). Masson's trichrome staining of the mouse liver tissues with or without mAb injection. Bars = 200 μm (left column). The procedure is shown below each figure. Measurement of hydroxyproline content (middle column) and αSMA(*Acta2*) expression (right column) in the livers. A full‐gel image of the αSMA western blotting is provided in supplementary material, Figure S3. (B) CCl_4_ treatment of mice with temporally inducible genetic loss of *Itga8* expression. The timing of Tam and CCl_4_ injections can be found at the top. Hydroxyproline content of *Itga8* ^flox/flox^;*Rosa26‐Cre* ^ER^ mice. Tam administration was identical for all groups. Cre‐recombination efficiency shown by red (non‐recombination) and green (post‐recombination) in vehicle or CCl_4_‐treated *mTmG* reporter mice livers. Fluorescence for mice carrying or not carrying the *Rosa26‐Cre* ^ER^ allele were compared (right). Bar = 100 μm. (C) α8 mRNA expression in human fibrotic liver samples by pathological fibrotic grading using RT‐qPCR. Backgrounds of the patients are shown in a flow chart (left) (*n* = 24 in F0, *n* = 38 in F1–3, and *n* = 24 in F4). Data were calculated by one‐way ANOVA followed by multiple comparison tests. Each dot represents an individual. Mean ± SEM (A,B) or medians and quartiles in (C).

The effects of neutralizing anti‐α8 mAb YZ3 [5] were evaluated in mouse models of cytotoxic (CCl_4_ treatment), NASH‐associated (choline‐deficient, l‐amino acid‐defined, high‐fat diet), and cholestatic (bile duct ligation) liver fibrosis. In all models, morphologic evidence of fibrosis was attenuated, and elevated hydroxyproline content and αSMA protein or mRNA expression were reduced by inhibition of α8β1 (Figure 2A and supplementary material, Figure S3), which were validated by quantification of the area stained by Masson's trichrome or αSMA (see supplementary material, Figure S4). We used CCl_4_ treatment in mice with global tamoxifen (Tam)‐inducible loss of α8 and found protection against increased hydroxyproline content. Cre‐recombination efficiency appeared to be excellent and unaffected by CCl_4_ treatment in *mTmG* reporter mice (Figure 2B) and the recombination was confirmed by identifying the expected version of α8 protein truncated by 69 residues by western blotting (see supplementary material, Figure S5). We then analyzed *ITGA8* expression in liver tissues from 90 human patients and found expression increased in fibrotic livers compared with F0 controls (*p* < 0.0001, Figure 2C).

### α8β1‐mediated myofibroblast differentiation

**Figure 3:**
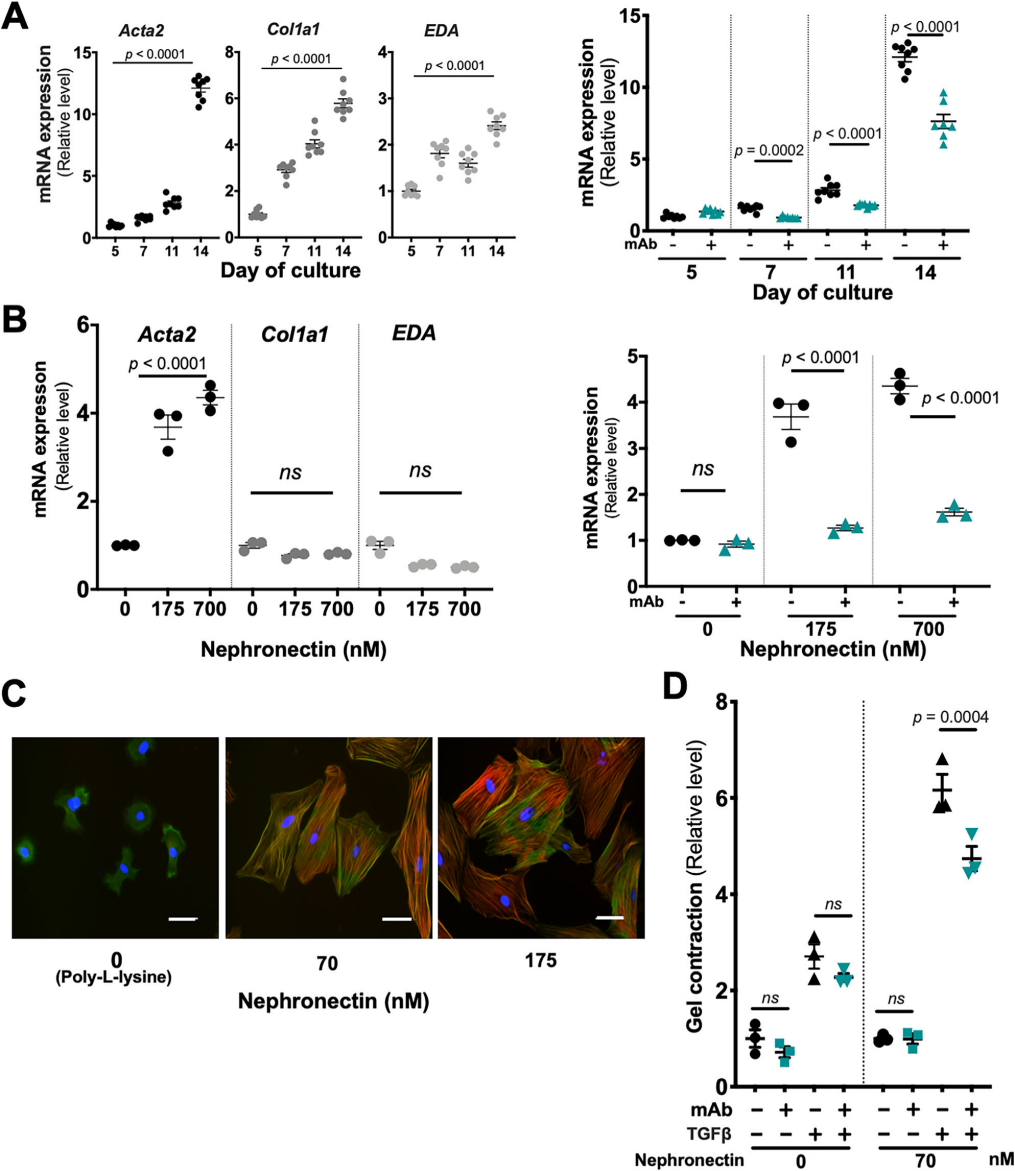
α8β1‐mediated myofibroblast differentiation of HSC/fibroblast. (A,B) Fibrotic markers, *Acta2*, *Col1a1*, and *EDA*, of rat HSCs (left) and effects of anti‐α8 mAb on *Acta2* (right) in 14‐day culture (A) or serum‐free 24 h culture on nephronectin‐coated plastic (B). Nephronectin non‐coated wells, with poly‐l‐lysine coating. (C) α8β1 engagement‐induced αSMA (red) and stress fiber formation (green) in rat fibroblasts. (D) Gel contraction of fibroblasts in the presence or absence of nephronectin. Data were calculated by one‐way ANOVA (A,B; left) with multiple comparison tests (D) or unpaired two‐tailed Student's *t*‐test (A,B; right). Each dot represents an animal (A,B) and replicated wells (D). Mean ± SEM.

To investigate the functional consequences of increased α8 for liver fibrosis, we inhibited or induced ligand engagement of α8β1 *in vitro*. HSCs grown on plastic upregulated fibrosis markers, *Acta2* (αSMA), *Col1a1* (collagen type I α1 chain) and *EDA* (fibronectin extra‐domain A), and *Acta2* was reduced by α8β1 inhibition (Figure 3A), whereas the inhibition had no effect on *Col1a1* and *EDA* (see supplementary material, Figure S6A). To explore the relevance of the *Acta2* induction to α8β1, HSCs were plated on to a ligand for α8β1, nephronectin, a basement membrane protein identified based on its function as an α8β1 ligand. α8β1 binds specifically only to nephronectin [13]. In serum‐free 24 h culture, *Acta2* was induced on nephronectin, and reduced by α8‐mAb, whereas expression of *Co1a1* or *EDA* was unaffected (Figure 3B). The ligand engagement‐induced *Acta2* expression and its inhibition by the mAb was recapitulated in fibroblasts (see supplementary material, Figure S6B). Plating the fibroblasts on nephronectin also induced αSMA immunofluorescence and formation of actin stress fibers (Figure 3C). Furthermore, nephronectin enhanced contraction of collagen gels by fibroblasts, which was reduced by α8 inhibition (*p* = 0.0004; Figure 3D). A collagen gel assay using the fibroblasts showed no contraction in 0.5% FCS medium, regardless of the presence of nephronectin, but with TGFβ (10 ng/ml) supplementation, contraction was detected and greatly enhanced by nephronectin, and this enhancement was reduced by α8 inhibition. The nephronectin concentrations used for each cell type were selected to be within the range of those required for specific interaction with α8β1 (see supplementary material, Figure S6C). These results indicate that α8β1‐mediated signal enhances fibroblast/HSC contractility and myofibroblast differentiation.

### TGFβ activation by α8β1 on HSCs

**Figure 4:**
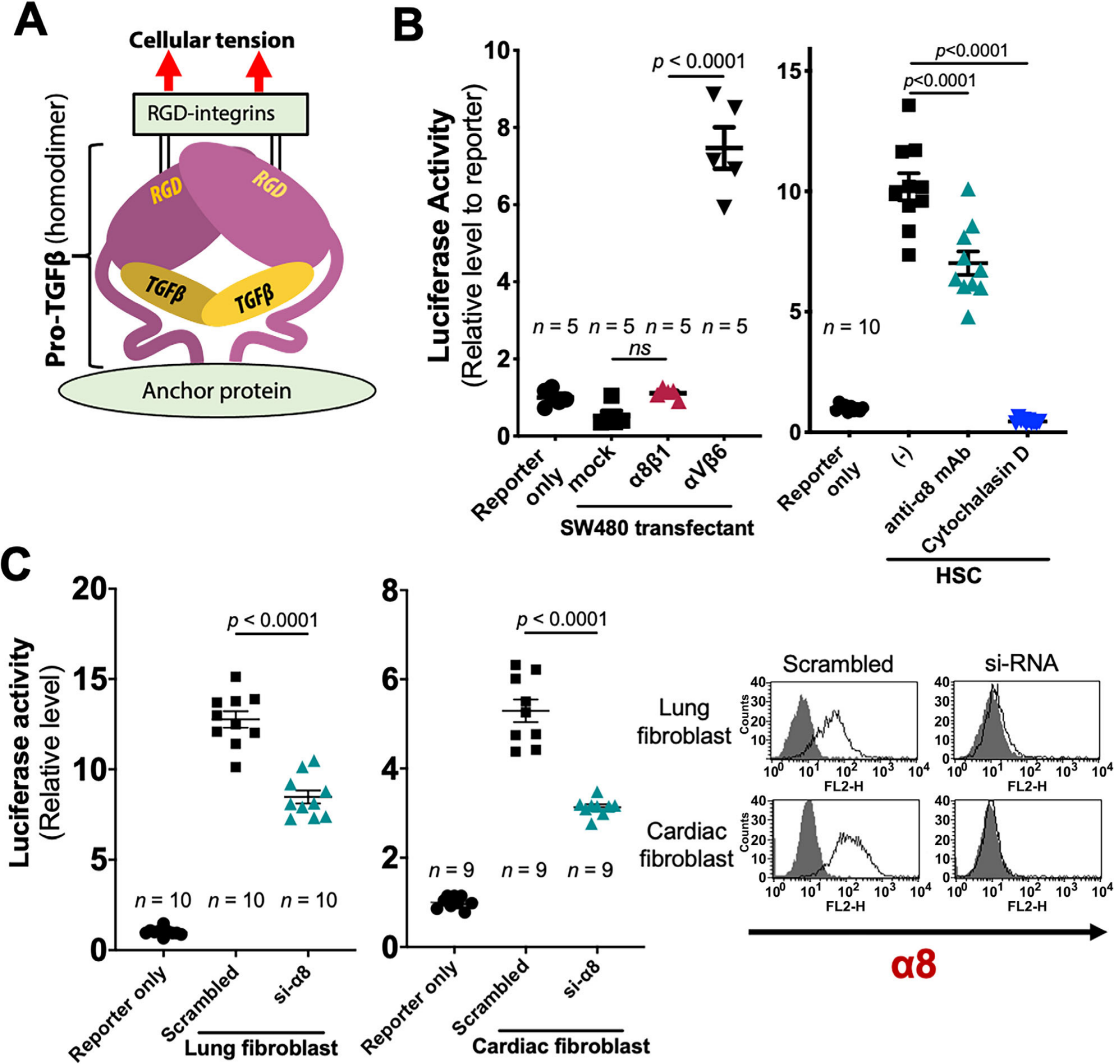
TGFβ activation by α8β1. (A) A schematic of a pro‐TGFβ heterodimer. Pro‐domains (purple) sequester mature TGFβs (yellow). (B) TGFβ activation of mock‐, α8‐, and β6‐transfected SW480 cells (left) and rat HSCs in the presence or absence of anti‐α8 mAb or cytochalasin D (right). (C) TGFβ activation by rat lung and cardiac fibroblasts treated by α8 targeting or control siRNA. α8 expression of the groups are shown in FACS histograms (right). Data were calculated by one‐way ANOVA followed by multiple comparison tests (B) or unpaired two‐tailed Student's *t*‐test (C). Each dot represents a replicated well. Mean ± SEM.

As several RGD‐binding integrins have been reported to activate TGFβ [14] (Figure 4A), we examined α8β1 for this ability. Although β6‐transfected SW480 cells clearly activated TGFβ, α8‐SW480 did not (Figure 4B, left). As, in contrast to αvβ6, α8β1 is expressed in fibroblasts, but not epithelial cells *in vivo* (Figure 1D), we employed α8‐expressing HSCs. Luciferase activity was detected and, notably, distinctly reduced with α8‐mAb by approximately 35% (*p* < 0.0001; Figure 3B, right). The TGFβ activation required actin polymerization as it was abolished by cytochalasin D, and we suspect is explained by much higher expression of *Acta2* in HSCs compared with SW480 (*p* < 0.0001; see supplementary material, Figure S7). Lung and heart fibroblasts expressing high levels of *Acta2* like HSCs, displayed α8β1‐mediated TGFβ activation (Figure 3C). α8β1 thus activates TGFβ when it is expressed by HSCs and fibroblasts.

## Discussion

HSC α8β1 integrin appears to contribute broadly to liver fibrosis as blockade or deletion of this integrin inhibits fibrosis in four different settings. *In vitro* studies suggest that α8β1 probably contributes to fibrosis by enhancing myofibroblast differentiation and by TGFβ activation of HSCs.

Our findings illuminated previously unknown characteristics of α8β1: *de novo* expression in activated HSCs *in vitro* and *in vivo*, upregulation of αSMA, and TGFβ activation on HSCs and fibroblasts. These findings fit well with previous observations of *Itga8* expression in other contractile cells, including airway [15] and gastric [16] smooth muscle cells, arrector pili [17] and sensory hair cells [18]. Recent single‐cell RNA‐sequencing data from NASH mice revealed exclusive *Itga8* expression in HSCs in liver cells [19]. Moreover, another group identified a class of murine HSCs that undergo myofibroblast differentiation, in which hierarchical clustering characterized the transition from a quiescent to a collagen‐producing phenotype, with upregulation of pro‐fibrogenic genes, including *Col1a1*, *Col1a2*, *Col3a1*, and *Lox* [20]. Interestingly, the upregulated genes include only one integrin, *Itga8*.

Cell matrix communication is characterized by redundant ligand–receptor interactions. The partial effects of anti‐α8 mAb found in this study for culture‐induced *Acta2* expression, gel contraction, and TGFβ activation could be attributed to contributions of other integrins. Nonetheless, inhibition of α8β1 *in vivo* potently inhibited liver fibrosis. The molecular mechanisms by which HSC activation leads to induction of *Itga8* and α8β1 engagement induces myofibroblast differentiation should be the focus of future studies.

The increased *ITGA8* expression in patients with hepatic fibrosis suggests that our findings are relevant to liver fibrosis in people. Because α8 expression is minimal in healthy liver, the relevance of this integrin as a driver of liver fibrosis has been largely overlooked. However, our findings that the specific expression of α8β1 in activated HSCs is critical for induction of a contractile phenotype and TGFβ activation make this integrin an attractive therapeutic target.

## Author contributions statement

NN, KT and YY designed the experiments. NN wrote the manuscript and conducted many of the experiments. KT generated and evaluated the knockout mice, performed the immunofluorescence experiments in mouse tissues, and wrote the manuscript. KK directed the induction of liver fibrosis in the mouse models. KS performed the pathological evaluation of fibrosis in mice. KC provided most of the human liver specimens and analyzed the patient data. TK provided some of the normal human liver specimens. DS verified the concept and data of this work and revised the manuscript. YY conceptualized this study and performed data analyses and interpretation and wrote the manuscript.

## Supporting information


**Supplementary materials and methods**

**Supplementary figure legends**
**Figure S1.** Specificity of anti‐α8 mAb YZ3
**Figure S2.** α8 expression in HSCs and fibroblasts
**Figure S3.** Full gel image of western blot for αSMA in Figure 2A (CCl_4_)
**Figure S4.** Measurement of fibrotic area in liver sections from three mouse models stained for collagen fibers and αSMA
**Figure S5.** Western blotting for the WT and mutant α8 in Tam‐inducible α8 knockout mice
**Figure S6.** Effects of α8β1 inhibition on *Col1a1* and *EDA*, and specificity of nephronectin to α8β1
**Figure S7.** RT‐qPCR for *Acta2*

**Table S1.** Antibodies used in this study
**Table S2.** PCR primer sequences
