# Supplementary material for: Induced hepatic stellate cell integrin, α8β1, enhances cellular contractility and TGFβ activity in liver fibrosis
Source: J Pathol. 2021 Feb 19;253(4):366–73. doi: 10.1002/path.5618 (PMC7986747; doi:10.1002/path.5618)
Supplement: Supplementary file 1 — Supplementary materials and methods [file PATH-253-366-s002.docx]

**Induced hepatic stellate cell integrin, α8β1, enhances cellular contractility and TGFβ activity in liver fibrosis**

N Nishimichi, K Tsujino *et al. J Pathol* DOI: 10.1002/path.5618

**Supplementary materials and methods**

Reference numbers refer to the main text list

**Cells and culturing**HSCs were isolated essentially as described previously [21]. In brief, the liver was perfused via the portal vein with PBS for 5 min and a buffer containing EGTA for 10 min then digested and homogenized in pronase E, collagenase D, and DNase I. HSCs were separated by specific gravity centrifugation (1700 x *g*) using 9% (w/v) Nycodenz. In a culture plate, isolated HSCs were incubated in DMEM containing 10% FBS, 100 U/ml penicillin, 100 μg/ml streptomycin, and 250 ng/ml amphotericin B in 5% CO_2_ at 37 °C. Non-adherent floating cells were removed during culture. Fibroblasts of the lung and heart were isolated from Wistar rats. Colon cancer cell line SW480 and hamster ovarian cancer cell line CHO were obtained from the American Type Culture Collection (ATCC, Manassas, VA, USA). Integrin β6-transfected [22] and α8-transfected [6] SW480 cells were described previously. All cells were cultured in DMEM containing 10% FBS, 100 U/ml penicillin, 100 μg/ml streptomycin, and 250 ng/ml amphotericin B in 5% CO_2_ at 37 °C, unless otherwise stated. Geneticin was added to cultures at 0.5 mg/ml of transfectants containing the neomycin resistance gene. Primary cultured line-expression data were retrieved from the FANTOM database [23].

**Antibodies**Chicken neutralizing anti-α8 mAb, YZ3 [17,24], and anti-α11, YW33, both of which react with human, mouse, and rat were prepared as described [6]. Specificity and the epitope of YZ3 are presented in Figure S5. The clone was screened by cell panning with α8- or α11-transfected SW480 cells in an scFv phage-display library. YZ3 was engineered into chimeric form with mouse IgG_1_κ-Fc for injection and *in vitro* use. Mouse IgG_1_ (Clone MOPC21) was used as an isotype control. In all *in vitro* experiments, YZ3 was used at a final concentration of 10 μg/ml, unless otherwise stated.

**Animals**For experimental liver fibrosis, female C57BL/6 mice (CLEA, Japan) were maintained in the animal barrier facility of Hiroshima University. The temporally inducible knockout mice were obtained by crossing *Itga8*^flox/flox^ [25] with *Rosa26-*Cre^ER^ mice [26] (Jackson Laboratory) at UCSF. Age- and sex-matched littermates were used as controls. Tamoxifen (Sigma-Aldrich) was injected twice to ensure recombination to all groups of the knockout mice. All mice were maintained under specific pathogen-free conditions. Eight-week-old mice were used in each experiment. Mice for the mAb injection and the genetic deletion were maintained at the animal facility of Hiroshima University and UCSF, respectively.

**RT-qPCR**Total RNA was extracted from cells and tissues and cDNA was synthesized using ReverTra Ace reverse transcription reagent (TOYOBO, Osaka, Japan). Quantitative PCR was performed using an ABI 7300 Real Time PCR system with SYBR FAST qPCR kit (Nippon Genetics, Tokyo, Japan). The target gene expression was normalized to *Rps18* mRNA expression. All primers used in this study (supplementary material, Table S2) were designed with Primer3 software. The 2^-ΔΔCt^ was calculated for relative expression of samples to a calibration sample for a gene normalized to *Rps18*.

**Flow cytometry**Cells were detached from the culture plates using 0.05% trypsin/0.53 mm EDTA and washed twice with serum-free DMEM. The cells were incubated with primary antibody followed by secondary antibody or with labeled primary mAb only. Data were acquired by FACSCalibur and analyzed using CellQuest Pro software (Becton Dickinson, Franklin Lakes, NJ, USA).

**Experimental fibrosis**In the hepatotoxic model, CCl_4_ (1 ml/kg) was injected subcutaneously in a 1:1 ratio with vehicle (olive oil). NASH mice were fed on a choline-deficient, l-amino acid-defined, high-fat diet (CDAHFD) [27] for 8 weeks. For the BDL model, the common bile duct was ligated with 4-0 silk sutures at two points. Anti-α8 mAb YZ3 was injected intraperitoneally twice per week at 25 mg/kg or 12.5 mg/kg as indicated in the figures. A total of 2.0–2.5 mg mAb was injected per mouse over the course of these experiments. The mAb concentration in serum at the end of the CCl_4_ experiments was 58–85 μg/ml.

**Hydroxyproline assay**

Liver tissue was hydrolyzed in 6 m HCl overnight at 110 °C. The lysate was centrifuged at 800 × *g* for 10 min and the supernatant filtered at 0.45 μm. Twenty microliters of the cleared supernatant was neutralized using 8 m NaOH, and 280 μl of distilled water was added, followed by 300 μl of isopropanol. The mixture was incubated with 100 μl of 0.84% chloramine-T in 42 mm sodium acetate, 2.6 mm citric acid, and 39.5% (vol/vol) isopropanol (pH 6.0) for 10 min at room temperature. The colorimetric reaction was developed by adding 500 μl of 0.248 g p-dimethyl amino benzaldehyde dissolved in 0.27 ml of 60% perchloric acid and 0.73 ml of isopropanol, then incubating for 90 min at 50 °C. Absorbance was measured at 570 nm. The hydroxyproline content of each sample was calculated from a standard curve using purified hydroxyproline (Sigma-Aldrich) as the standard.

**Measurement of areas stained by Masson’s trichrome or αSMA immunostaining**Sections cut at 5 μm from paraffin blocks were stained with Masson’s trichrome or the anti-αSMA antibody (Agilent-DAKO). Stained area was quantified using ImageJ (2.1.0) software (https://imagej.net).

**Human liver tissues**Human samples were obtained from surgically resected liver tissue at Hiroshima University Hospital. Non-cancerous tissue was collected from patients with tumors and the absence of cancer cell invasion was verified pathologically. The degree of liver fibrosis was assessed according to the scoring system of Desmet [28] using a scale of 0–4 (F0: Absent; F1: mild fibrosis with periportal fibrous expansion; F2: moderate fibrosis with portal-portal septa (≥1 septum); F3: severe fibrosis with porto-central septa (≥1 septum); F4: cirrhosis).

**Recombinant nephronectin proteins**A nephronectin fragment corresponding to amino acids 378–403 [14] was expressed as a glutathione *S*-transferase fusion protein in *E. coli* with pGEX6P plasmid as described previously [29].

**Western blotting**Tissues were lysed for 30 min at 4 °C using a buffer containing 1% aprotinin, 1% PMSF, and a protease inhibitor cocktail with gentle agitation. Debris was removed by centrifugation at 12 000 x *g* for 15 min. The protein concentration in supernatants was determined using the Bicinchoninic Acid Protein Assay kit (Sigma-Aldrich). After boiling for 5 min, samples were loaded onto a 5–20% polyacrylamide gradient gel for electrophoresis under reducing or non-reducing (for integrins) condition. Proteins were transferred to a PVDF membrane, probed with specific antibodies overnight at 4°C with gentle shaking, and detected with HRP-conjugated secondary antibodies and Luminata Forte HRP-oxidization substrate. (Merk, Tokyo, Japan) Chemiluminescence was visualized with a blot scanner and quantified using Image Studio software (LI-COR, Lincoln, NE, USA). Expression levels of the target molecule were normalized to β-tubulin then compared to the values from control mice.

**Immunofluorescence**For immunofluorescence, sections of mouse tissues were deparaffinized and rehydrated then heated in citrate buffer (10 mm, pH 6.0) and blocked with 10% normal serum and 0.5% BSA. Alexa 488- or Cy3-conjugated primary antibodies were applied to sections for 1 h at room temperature. For immunofluorescence to study stress fiber formation in cultured cells, serum starved rat lung fibroblasts (2 × 10^4^) were seeded onto cell imaging slides coated with nephronectin fragment at 70 or 175 nm or poly-l-lysine (Sigma-Aldrich) and incubated for 24 h in serum-free conditions. Cells were fixed and permeabilized with 0.1% (w/v) Triton X-100 for 5 min. After blocking, F-actin and αSMA were stained with phalloidin (cytoskeleton) and the antibody (Sigma-Aldrich), respectively. Nuclei were counterstained with DAPI.

**Gel contraction assay**Rat lung fibroblasts were serum-starved in DMEM with 0.5% FBS for 48 h, and 2 × 10^5^ cells were suspended in 550 μl of collagen I (2.33 mg/ml) gel solution (Corning, NY, USA) with or without recombinant nephronectin fragment (70 nm) or anti-α8β1 blocking mAb (10 μg/ml). The gel was polymerized at the bottom of a well in a 24-well plate and incubated at 37 °C for 72 h in 1 ml of the 0.5% FBS DMEM supplemented with or without 10 ng/ml of recombinant human TGF-β1 (Peprotech Cranbury, NJ, USA). The gel was then carefully released from the bottom of the well, and the diameter was measured after 8 h using ImageJ software for calculation of the contraction normalized to a control without nephronectin, TGFβ or the mAb.

**TGFβ activation assay**TGFβ activation by integrins was measured with the luciferase assay (Promega, Madison, WI, USA) using luciferase reporter cells mixed with test cells. The reporter cells used were either mink lung epithelial cells stably transfected with a plasmid containing luciferase cDNA downstream of a TGFβ-sensitive portion of the plasminogen activator inhibitor 1 promoter (TMLC) [5] or a Smad-binding (CAGA)_9_ sequence from the adenovirus major late promoter region (CAGA9-Luc) [30]. HSCs (10 days of culture), or lung or cardiac fibroblasts (7 days of culture) were examined. Following incubation for 16 h at 37 °C in DMEM with 10% FBS, luciferase activity was measured using TriStar LB941 luminometer (Berthold, Bad Wildbad, Germany). Higher luciferase activity than the original TMLC reporter cells [5] was confirmed in each experiment.

**Statistical analyses**
Statistical analyses were performed using Prism 8 (GraphPad, San Diego, CA, USA). Data are presented as the mean ± SEM or in Tukey’s box and whisker plots with upper and lower quartiles. For comparisons between two groups, an unpaired two-tailed Student’s *t*-test or a two-tailed Mann–Whitney *U* test was applied. Student’s *t-*test was applied to data with a normal distribution, else a two-tailed Mann–Whitney test was performed. Normality was computed by the D’Agostino-Person omnibus normality test or Shapiro–Wilk’s test, where applicable. When comparing three or more groups, one-way ANOVA was used, followed by Tukey’s (each mean with every other mean) or Dunnett’s (a mean of control with every other mean), and Sidak’s (selected pairs of means) multiple-comparison tests. Before the one-way ANOVA analysis, the absence of differences in SD between groups was established using the Brown-Forsythe test. Correlations were assessed by linear regression analysis using Pearson’s correlation. Outliers were determined by the ROUT method [31] with alpha 0.05. Each experiment was repeated at least three times. All data for dot plots are represented as the mean ± SEM, unless otherwise stated.
