# Supplementary material for: Induced hepatic stellate cell integrin, α8β1, enhances cellular contractility and TGFβ activity in liver fibrosis
Source: J Pathol. 2021 Feb 19;253(4):366–73. doi: 10.1002/path.5618 (PMC7986747; doi:10.1002/path.5618)
Supplement: Supplementary file 2 — Supplementary figure legends Figure S1. Specificity of anti‐α8 mAb YZ3 Figure S2. α8 expression in HSCs and fibroblasts Figure S3. Full gel image of western blot for αSMA in Figure 2A (CCl4) Figure S4. Measurement of fibrotic area in liver sections from three mouse models stained for collagen fibers and αSMA Figure S5. Western blotting for the WT and mutant α8 in Tam‐inducible α8 knockout mice Figure S6. Effects of α8β1 inhibition on Col1a1 and EDA, and specificity of nephronectin to α8β1 Figure S7. RT‐qPCR for Acta2 [file PATH-253-366-s001.zip › path5618-sup-0002-SuppFigLegsS1-S7.docx]

**Induced hepatic stellate cell integrin, α8β1, enhances cellular contractility and TGFβ activity in liver fibrosis**

N Nishimichi, K Tsujino *et al. J Pathol* DOI: 10.1002/path.5618

**Supplementary figure legends S1–S7**

Reference numbers refer to the main text list

**Figure S1. Specificity of anti-α8 mAb YZ3.** (A) FACS histograms showing recognition of α8 (red box) and no recognition of the other l-domain less integrin α subunits (blue boxes) by YZ3. Each box contains results from one cell line, whose integrin expressions are shown in the right of histograms. No recognition of the cell lines by YZ3 is presented on the bottom of each blue boxes. Nine mAbs used in this FACS analyses are summarized in supplementary material, Table S1. (B) Epitope sequences in human α8 for YZ3 is aligned with that of mouse, rat, chicken, and platypus. Residues in red are divergent from mammals and asterisks indicate conserved residues. In mammals, this epitope sequence is fully conserved [4].

**Figure S2. α8 expression in HSCs and fibroblasts.** (A) A rat counterpart of Figure 1A. Data were analyzed by one-way ANOVA and presented as mean ± SEM (*n* = 3 animals). (B) Increase in α8 protein expression by FACS during culture activation, through day 1 to 14. Changes in α8 expression by MFI is presented as a line graph below. (C) FACS histogram of α8 protein in primary rat lung, heart, and kidney fibroblasts.

**Figure S3. Full gel image of western blot for αSMA in Figure 2A (CCl4).** Expression of α-SMA in CCl_4_-treated mice (Figure 2A, top) was quantified from band intensity of western blotting, normalized to β-tubulin. Two primary antibodies were mixed for the probing. A representative full-gel image (vehicle, *n* = 2; IgG, *n* = 3; mAb, *n* = 3) is shown from pooled data from three experiments.

**Figure S4. Measurement of fibrotic area in liver sections from three mouse models stained for collagen fibers and αSMA.** Digital image analysis of Masson’s trichrome staining (left column) and α-SMA immunohistochemistry (right column) from mice with anti-α8 mAb or control IgG injection. Percentages of stained area are shown in Tukey’s Box and Whiskers plot. Data are calculated by unpaired two-tailed Student’s *t*-tests. “n” in each graph indicates the number of animals in the group.

**Fig. S5. Western blotting for the WT and mutant α8 in Tam-inducible α8 knockout mice.** The loxP sites flank exon 29 and 30 encoding extracellular, transmembrane, and the cytoplasmic domain of α8 subunit, consisting of 14, 27, and 28 amino acids, respectively. The difference in the mobility between WT and mutant derived from 69 amino acids in Val994-Ala1062. The primary antibody used (AF4076) was raised against synthetic peptide corresponding to α8 entire extracellular domain (Phe38-Phe1007). GAPDH was used as a loading control.

**Figure S6. Effects of α8β1 inhibition on Col1a1 and EDA, and specificity of nephronectin to α8β1.** (A) Changes in *Col1a1* and *EDA* in culture activation of HSCs for 14 days in the presence or absence of anti-α8 mAb. (B) Increase in *Acta2* expression of lung fibroblasts dependently of on nephronectin concentrations, and reduction of the increase by anti-α8 mAb. (C) Cell adhesion of HSCs (left) and fibroblasts (right) on varying concentration of nephronectin. The difference in adhesion in the presence and absence of anti-α8 mAb (black and red, respectively) indicates specific interaction of nephronectin with α8β1 in each cell line. Data are calculated by unpaired two-tailed Student’s *t*-tests. Each dot represents replicated wells (A,B) and mean of five wells (C). Mean ± SEM.

**Figure S7.** **RT-qPCR for *Acta2*.** (A) *Acta2* mRNA expression of SW480 cells and rat HSCs, lung, and cardiac fibroblasts with *Rps18* as a reference gene. (B) Comparable expression level of human *Rsp18* in human colon cancer cell lines Caco-2 and COLO-320, fibroblasts, and HSCs in the FANTOM database.
