# Supplementary figures and images for: Induced hepatic stellate cell integrin, α8β1, enhances cellular contractility and TGFβ activity in liver fibrosis
Source: J Pathol. 2021 Feb 19;253(4):366–73. doi: 10.1002/path.5618 (PMC7986747; doi:10.1002/path.5618)

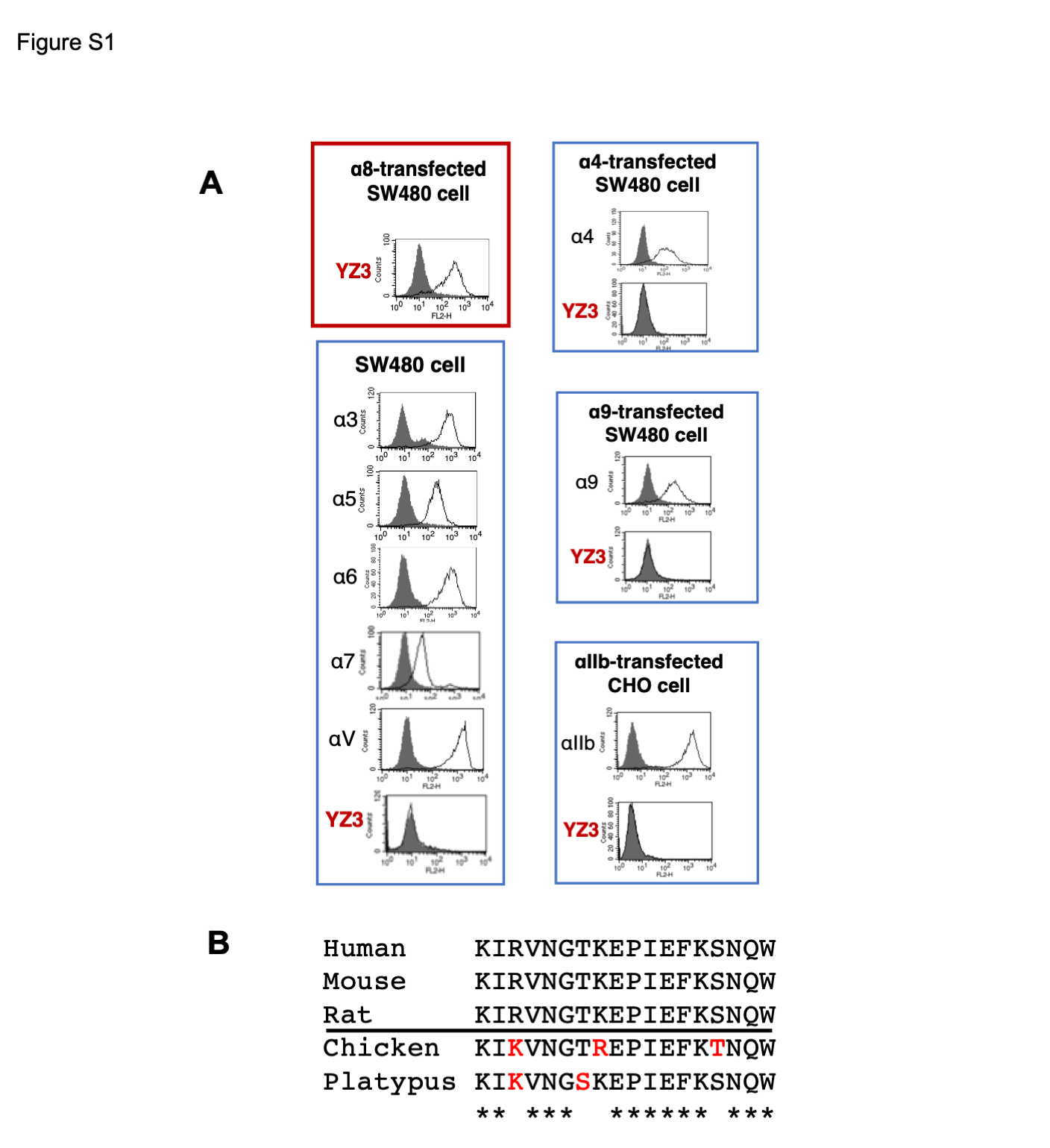

Supplement: Supplementary file 2 — Supplementary figure legends Figure S1. Specificity of anti‐α8 mAb YZ3 Figure S2. α8 expression in HSCs and fibroblasts Figure S3. Full gel image of western blot for αSMA in Figure 2A (CCl4) Figure S4. Measurement of fibrotic area in liver sections from three mouse models stained for collagen fibers and αSMA Figure S5. Western blotting for the WT and mutant α8 in Tam‐inducible α8 knockout mice Figure S6. Effects of α8β1 inhibition on Col1a1 and EDA, and specificity of nephronectin to α8β1 Figure S7. RT‐qPCR for Acta2 [file PATH-253-366-s001.zip › path5618-sup-FigureS1.tif]

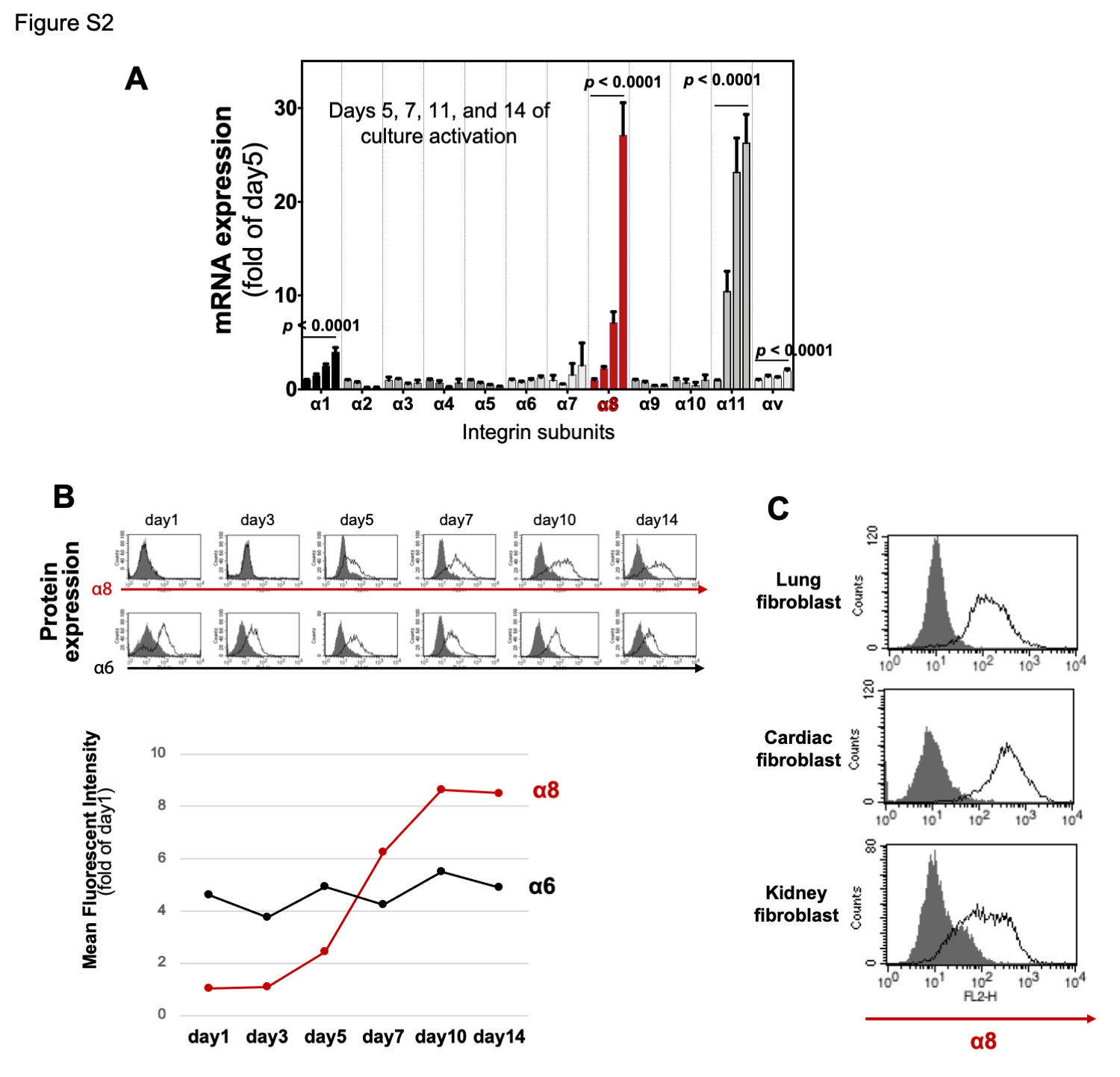

Supplement: Supplementary file 2 — Supplementary figure legends Figure S1. Specificity of anti‐α8 mAb YZ3 Figure S2. α8 expression in HSCs and fibroblasts Figure S3. Full gel image of western blot for αSMA in Figure 2A (CCl4) Figure S4. Measurement of fibrotic area in liver sections from three mouse models stained for collagen fibers and αSMA Figure S5. Western blotting for the WT and mutant α8 in Tam‐inducible α8 knockout mice Figure S6. Effects of α8β1 inhibition on Col1a1 and EDA, and specificity of nephronectin to α8β1 Figure S7. RT‐qPCR for Acta2 [file PATH-253-366-s001.zip › path5618-sup-FigureS2.tif]

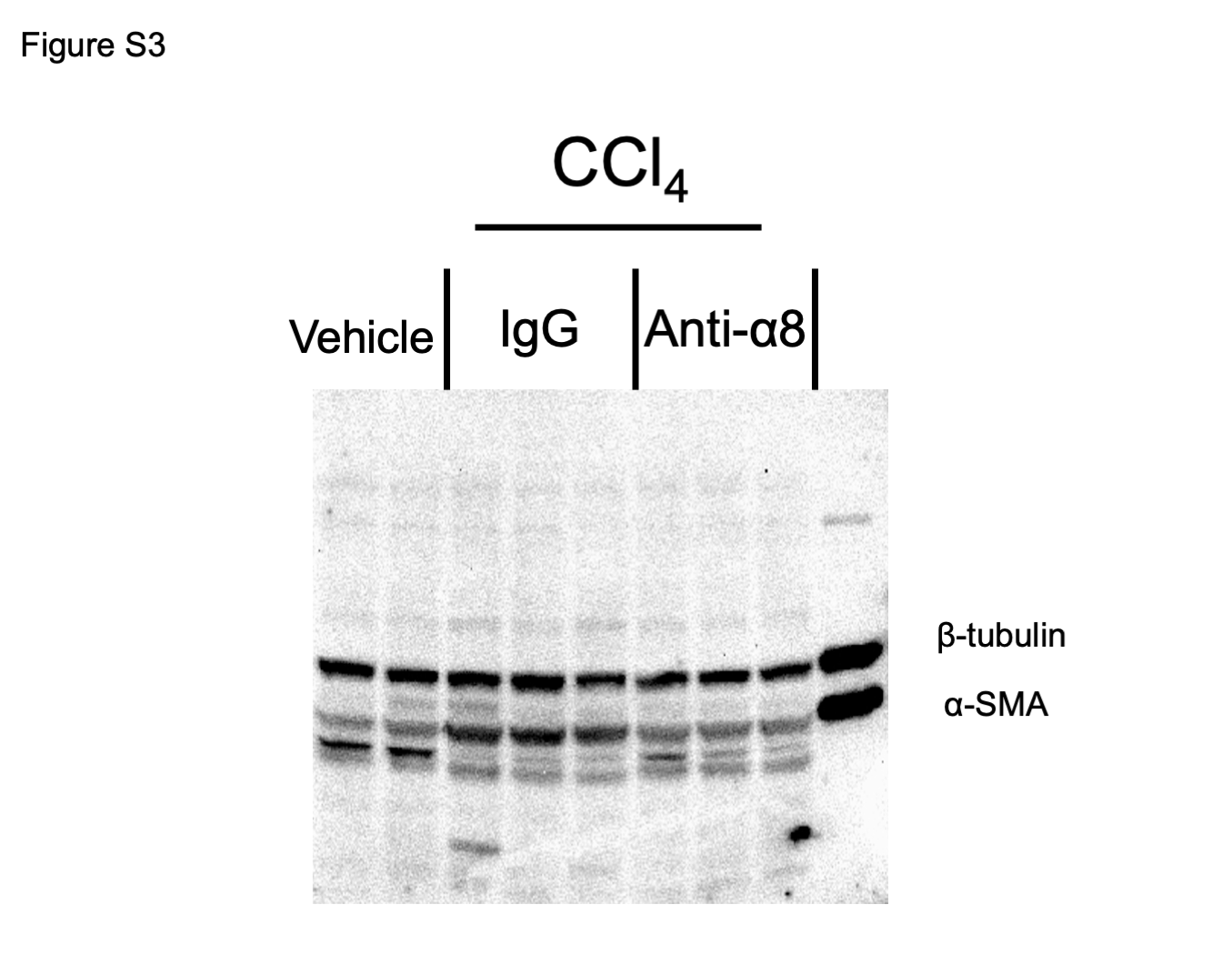

Supplement: Supplementary file 2 — Supplementary figure legends Figure S1. Specificity of anti‐α8 mAb YZ3 Figure S2. α8 expression in HSCs and fibroblasts Figure S3. Full gel image of western blot for αSMA in Figure 2A (CCl4) Figure S4. Measurement of fibrotic area in liver sections from three mouse models stained for collagen fibers and αSMA Figure S5. Western blotting for the WT and mutant α8 in Tam‐inducible α8 knockout mice Figure S6. Effects of α8β1 inhibition on Col1a1 and EDA, and specificity of nephronectin to α8β1 Figure S7. RT‐qPCR for Acta2 [file PATH-253-366-s001.zip › path5618-sup-FigureS3.tif]

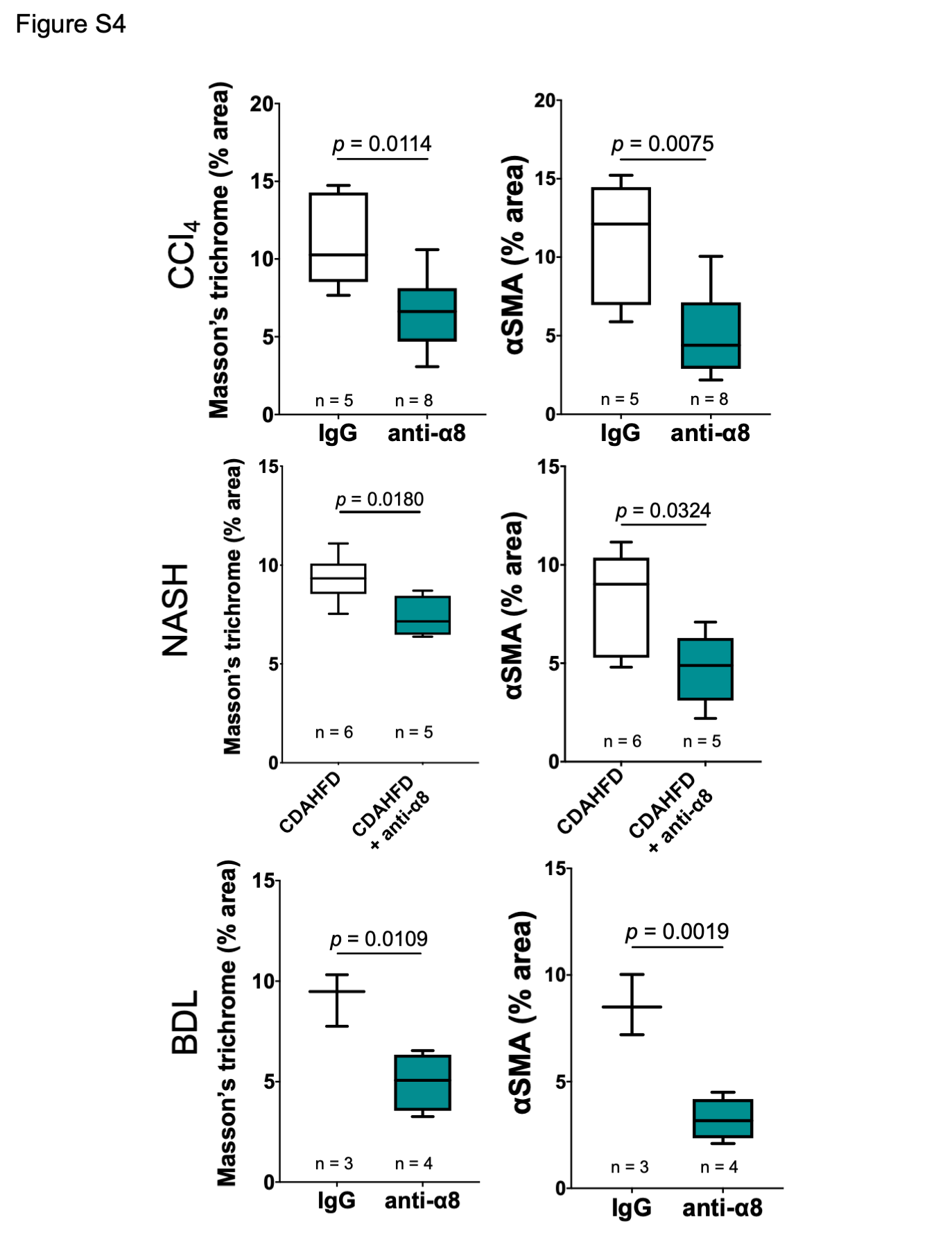

Supplement: Supplementary file 2 — Supplementary figure legends Figure S1. Specificity of anti‐α8 mAb YZ3 Figure S2. α8 expression in HSCs and fibroblasts Figure S3. Full gel image of western blot for αSMA in Figure 2A (CCl4) Figure S4. Measurement of fibrotic area in liver sections from three mouse models stained for collagen fibers and αSMA Figure S5. Western blotting for the WT and mutant α8 in Tam‐inducible α8 knockout mice Figure S6. Effects of α8β1 inhibition on Col1a1 and EDA, and specificity of nephronectin to α8β1 Figure S7. RT‐qPCR for Acta2 [file PATH-253-366-s001.zip › path5618-sup-FigureS4.tif]

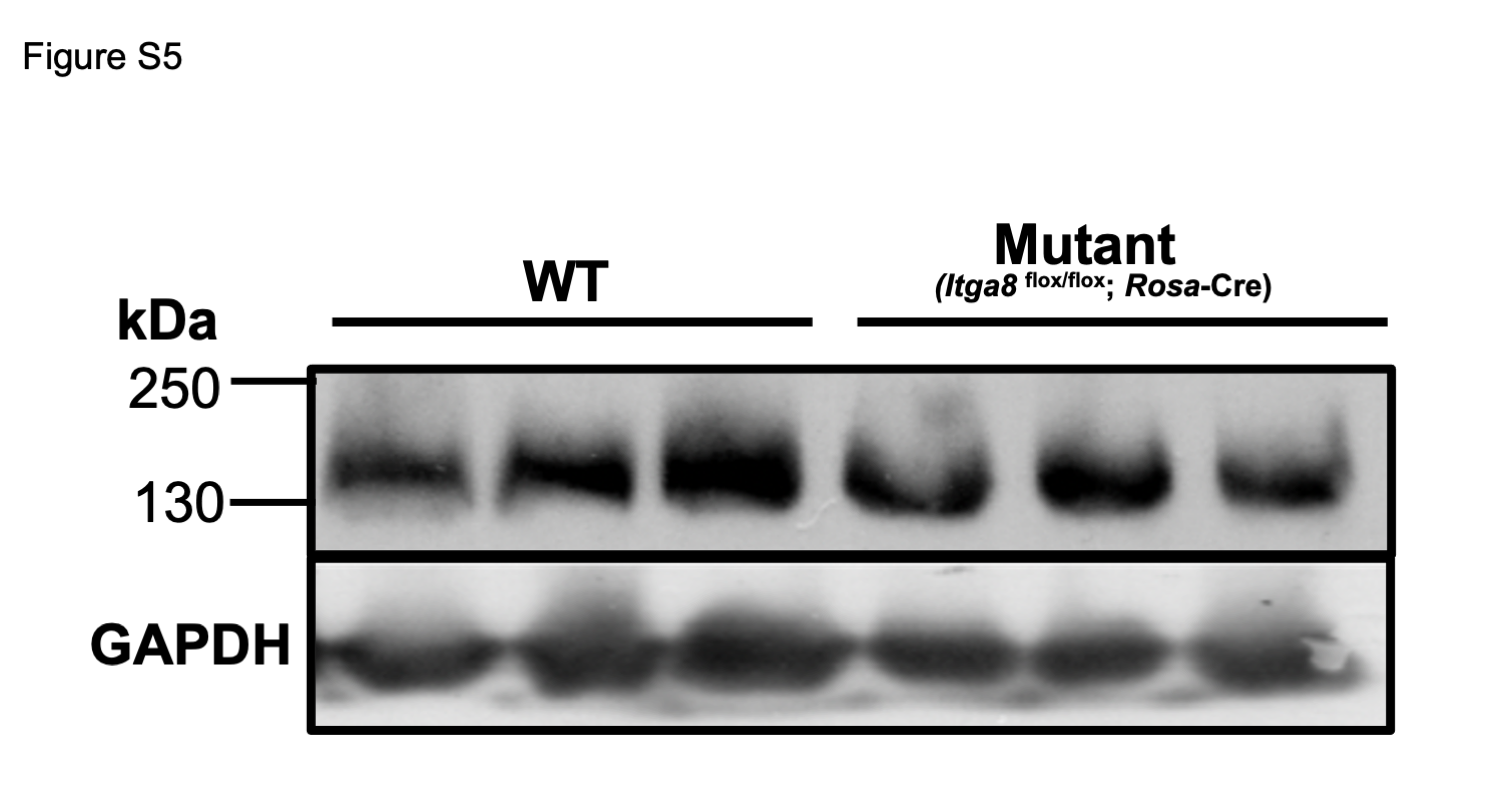

Supplement: Supplementary file 2 — Supplementary figure legends Figure S1. Specificity of anti‐α8 mAb YZ3 Figure S2. α8 expression in HSCs and fibroblasts Figure S3. Full gel image of western blot for αSMA in Figure 2A (CCl4) Figure S4. Measurement of fibrotic area in liver sections from three mouse models stained for collagen fibers and αSMA Figure S5. Western blotting for the WT and mutant α8 in Tam‐inducible α8 knockout mice Figure S6. Effects of α8β1 inhibition on Col1a1 and EDA, and specificity of nephronectin to α8β1 Figure S7. RT‐qPCR for Acta2 [file PATH-253-366-s001.zip › path5618-sup-FigureS5.tif]

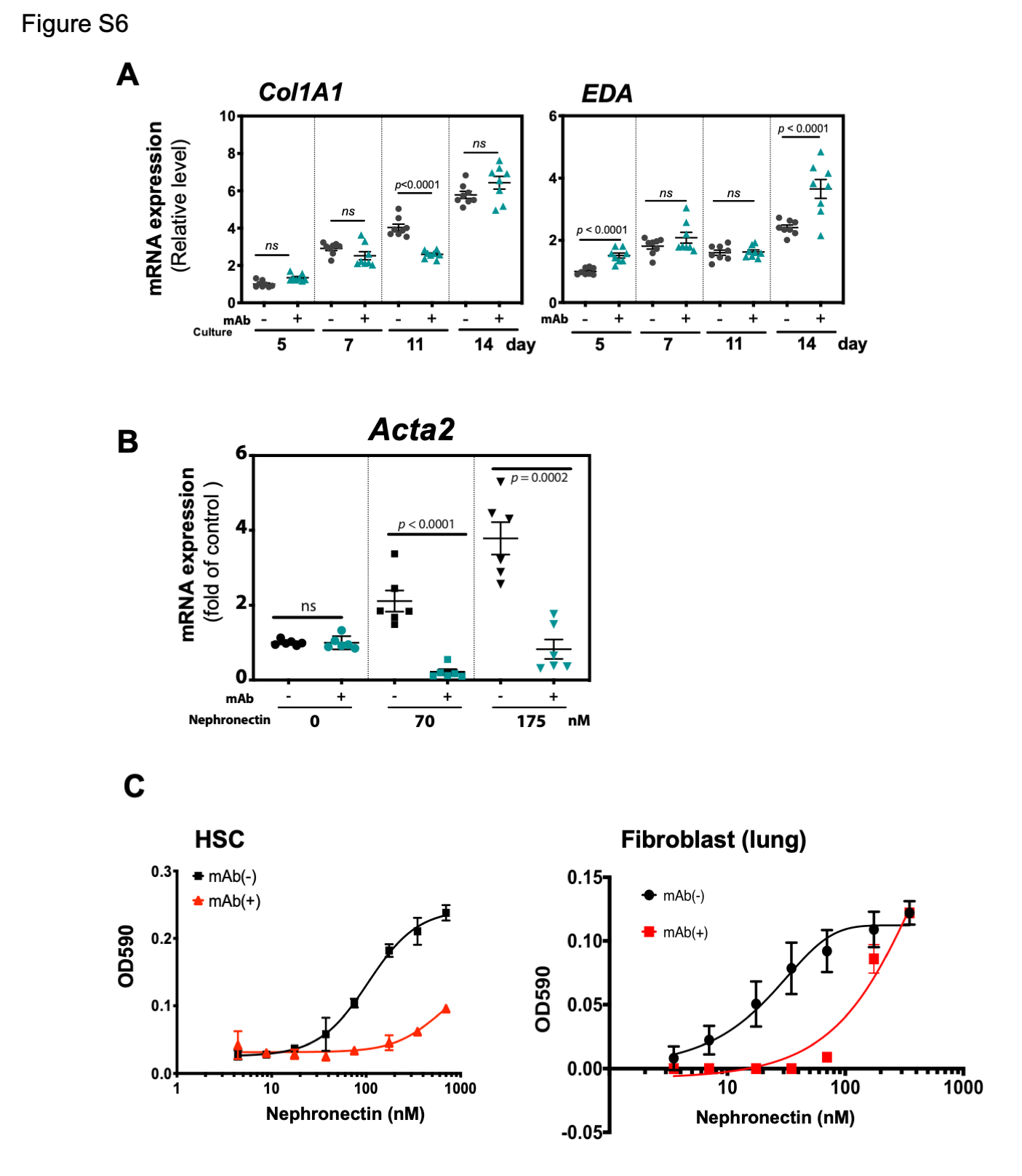

Supplement: Supplementary file 2 — Supplementary figure legends Figure S1. Specificity of anti‐α8 mAb YZ3 Figure S2. α8 expression in HSCs and fibroblasts Figure S3. Full gel image of western blot for αSMA in Figure 2A (CCl4) Figure S4. Measurement of fibrotic area in liver sections from three mouse models stained for collagen fibers and αSMA Figure S5. Western blotting for the WT and mutant α8 in Tam‐inducible α8 knockout mice Figure S6. Effects of α8β1 inhibition on Col1a1 and EDA, and specificity of nephronectin to α8β1 Figure S7. RT‐qPCR for Acta2 [file PATH-253-366-s001.zip › path5618-sup-FigureS6.tif]

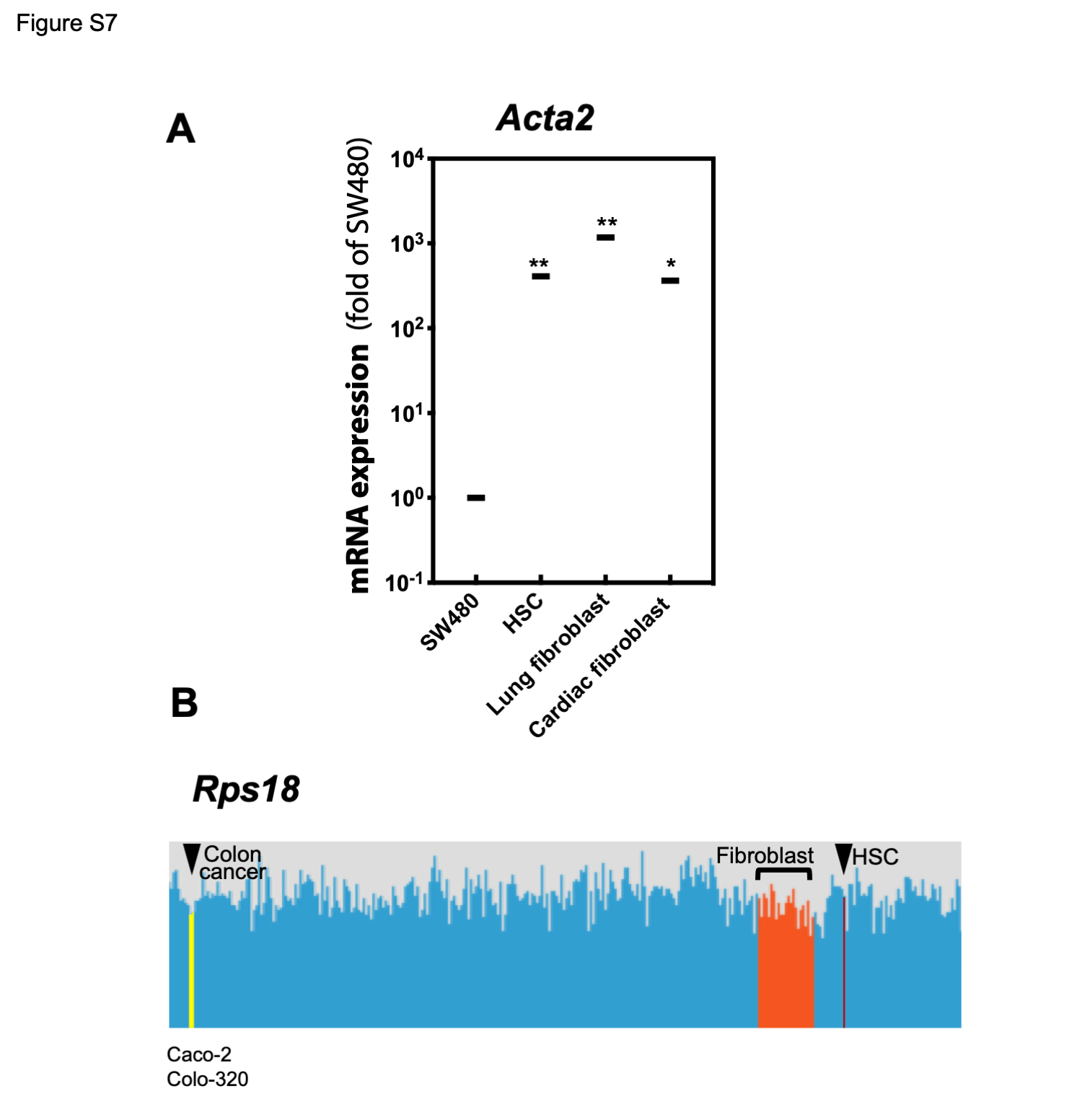

Supplement: Supplementary file 2 — Supplementary figure legends Figure S1. Specificity of anti‐α8 mAb YZ3 Figure S2. α8 expression in HSCs and fibroblasts Figure S3. Full gel image of western blot for αSMA in Figure 2A (CCl4) Figure S4. Measurement of fibrotic area in liver sections from three mouse models stained for collagen fibers and αSMA Figure S5. Western blotting for the WT and mutant α8 in Tam‐inducible α8 knockout mice Figure S6. Effects of α8β1 inhibition on Col1a1 and EDA, and specificity of nephronectin to α8β1 Figure S7. RT‐qPCR for Acta2 [file PATH-253-366-s001.zip › path5618-sup-FigureS7.tif]
