# Supplementary material for: Induced hepatic stellate cell integrin, α8β1, enhances cellular contractility and TGFβ activity in liver fibrosis
Source: J Pathol. 2021 Feb 19;253(4):366–73. doi: 10.1002/path.5618 (PMC7986747; doi:10.1002/path.5618)
Supplement: Supplementary file 3 — Table S1. Antibodies used in this study Table S2. PCR primer sequences [file PATH-253-366-s003.docx]

**Induced hepatic stellate cell integrin, α8β1, enhances cellular contractility and TGFβ activity in liver fibrosis**

N Nishimichi, K Tsujino *et al. J Pathol* DOI: 10.1002/path.5618

**Supplementary tables S1 and S2**

**Table S1. Antibodies used in this study**

***Primary antibody***

| **Anti-** | **Epitope** | **Antigen species** | **Immunogen** | **React with** | **Host** | **Clone** | **Block** | **Number** | **Company** | **Used in** | **Dilution** |  |
| --- | --- | --- | --- | --- | --- | --- | --- | --- | --- | --- | --- | --- |
| **Monoclonal** | | | | | | | | | | | | |
| **α3** | Extracellular | Human | Human transfectant | Human | Mouse | IA3 | Yes | ab20141 | Abcam  (Cambridge, UK) | FACS | 1:200 |  |
| **α4** | Extracellular | Human | T-cell | Human, Monkey | Mouse | P1H4 | ND | MAB16983 | Merk  (Tokyo, Japan) | FACS | 1:200 |  |
| **α5** | Extracellular | Human | Jurkat cells | Human, Monkey | Mouse | JBS5 | Yes | MAB1969 | Merk | FACS | 1:200 |  |
| **α6** | Extracellular | Human | SW1222 | Human | Mouse | 4F10 | ND | ab20142 | Abcam | FACS | 1:200 |  |
| **α7** | Extracellular | Mouse | Myoblast | Human, Mouse | Mouse | 3C12 | ND | ab4069 | Abcam | FACS | 1:200 |  |
| **α8** | 80 KIRVNGT 89 | Mouse | Chicken transfectant | Human, Mouse, Rat, others | Chicken | YZ3 | Yes | NA | In house | FACS, Block | 1:240 |  |
| **α9** | Extracellular | Mouse | Chicken transfectant | Human, Mouse, Rat, others | Chicken | YA4 | Yes | NA | In house | FACS | 1:200 |  |
| **α11** | I-domain | Mouse | Recombinant protein | Human, Mouse, Rat, others | Chicken | YW33 | Yes | NA | In house | FACS | 1:1,000 |  |
| **αV** | β-propeller | Human | Endothelial cells | Human | Mouse | L230 | Yes | HB8448 | ATCC  (Manassas, VA, USA) | FACS | 1:60 |  |
| **αIIb** | ND | Human | Purified protein | Human | Mouse | HIP8 | ND | ab195959 | Abcam | FACS | 1:100 |  |
| **α6** | Extracellular | Rat | Esophageal cell line | Rat | Mouse | mAb5A | ND | MAB1410 | OriGene (Rockville, MD, USA) | FACS | 1:10 |  |
| **βtubulin** | N-terminus | ND | Synthetic peptide | Human, Mouse, Rat, others | Rabbit | 9F3 | ND | #2128 | Cell Signaling Technology  (Danvers, MA, USA) | Western  blotting | 1:1,000 |  |
| **αSMA** | N-terminus | Human | Synthetic decapeptide | Human, Mouse, Rat, others | Mouse | 1A4 | ND | M0851 | Agilent-DAKO  (Santa Clara, CA, USA) | Histo-chemistry | 1:100 |  |
| **αSMA** | N-terminus | Human | Synthetic decapeptide | Human, Mouse, Rat, others | Mouse | 1A4 | ND | A5228 | Sigma-Aldrich (St Louis, MO, USA) | Fluorescence | 1: 2,000 |  |
| **PDGFRβ** | ND | Mouse | No information | Human, Mouse | Rat | ABP5 | Yes | 14-1402 | Thermo Fisher (Tokyo, Japan) | Fluorescence | 1:100 |  |
| **Polyclonal** | | | | | | | | | | | | |
| **α8** | Extracellular | Mouse | Synthetic peptide 38-1007 | Human, Mouse, | Goat | NA | ND | AF4076 | R&D  (Minneapolis, MN, USA) | Western | 1:500 |  |
| **GAPDH** | ND | Mouse | Synthetic peptide 314-333 | Human, Mouse, Rat, others | Rabbit | NA | ND | G9545 | Sigma-Aldrich | Western | 1: 20,000 |  |

***Secondary antibody***

| Antigen | Antigen species | Host | Conjugation | Catalogue | Company |
| --- | --- | --- | --- | --- | --- |
| IgG | Mouse | Goat | PE | 405307 | BioLegend  (San Diego, CA, USA) |
| IgG | Rabbit | Donkey | HRP | 406401 | BioLegend |
| IgG | Goat | Rabbit | HRP | Discontinued | SantaCruz Biotechnology  (Dallas, TX, USA) |
| IgG (H+L) | Mouse | Goat | Alexa Fluor 546 | A11030 | Thermo Fisher |

***Isotype control antibody***

| **Immunogen** | **Host** | **Clone** | **Catalogue** | **Company** |
| --- | --- | --- | --- | --- |
| No information | Mouse | MOPC21 | BE0083 | Bio X Cell  (Lebanon, NH, USA) |

**Table S2. PCR primer sequences**

| **Species** | **Gene** | **Forward** | **Reverse** |
| --- | --- | --- | --- |
| **Rat** | *Itga1* | 5’-AAAGAGGAACAGGGCAAGGT-3’ | 5’-CCATCCACGTTGAGGTCTTT-3’ |
|  | *Itga2* | 5’-CTTGGTAGGTGCTCCCATGT-3’ | 5’-GCCATCCATGTTGATGTCTG-3’ |
|  | *Itga5* | 5’-GCAGCTTCCCCAAAAGAAAC-3’ | 5’-TGTAGAGGACGTAGATGAGCAG-3’ |
|  | *Itga6* | 5’-GCCAAAACAGTGCAGACAGA-3’ | 5’-GGATGCCTTTCTGAATTGGA-3’ |
|  | *Itga8* | 5’-CCAAGACCCACAGGTCCTTA-3’ | 5’-CTGGCGTTTCCGTTGTAAAT-3’ |
|  | *Itga9* | 5’-CACTGGTGCATGAAGTGGAC-3’ | 5’-TGTTGTAGACCTGGAGAGTG-3’ |
|  | *Itga11* | 5’-GGCCTCCATTCAACTGTGTT-3’ | 5’-TGGCTCAGGTCTTCCTCAGT-3’ |
|  | *Itgav* | 5’-GTTCCAAGAGCAGCAAGGAC-3’ | 5’-CAGAGGGGCTCCAATAAACA-3’ |
|  | *Acta2* | 5’-GACAATGGCTCCGGGCTCTGT-3’ | 5’-TGCCGTGTTCTATCGGATAC-3’ |
|  | *Col1a1* | 5’-TAAAGGGTCATCGTGGCTTC-3’ | 5’-ACCGTTGAGTCCATCTTTGC-3’ |
|  | *Fn-eda* | 5’-ATTGACCGCCCTAAAGGACT-3’ | 5’-CAAGGCAACCACACTGACTG-3’ |
|  | *Rps18* | 5’-TGTGGTGTTGAGGAAAGCAG-3’ | 5’-TGGCCAGAACCTGGCTATAC-3’ |
| **Mouse** | *Acta2* | 5’-AATGGCTCTGGGCTCTGTAA-3’ | 5’-CTCTTGCTCTGGGCTTCATC-3’ |
|  | *Col1a1* | 5’-GAGCGGAGAGTACTGGATCG-3’ | 5’-TACTCGAACGGGAATCCATC-3’ |
|  | *Gapdh* | 5’-TGTGTCCGTCGTGGATCTGA-3’ | 5’-TTGCTGTTGAAGTCGCAGGAG-3’ |
| **Human** | *ITGA8* | 5’-TTGTGAGCCTGCAAATCAAC-3’ | 5’-CAGGATGGTGTCACTGATGG-3’ |
|  | *RSP18* | 5’-GAGGATGAGGTGGAACGTGT-3’ | 5’-TCTTCAGTCGCTCCAGGTCT-3’ |
|  | *ACTA2* | 5'-TTCAATGTCCCAGCCATGTA-3’ | 5'-GAAGGAATAGCCACGCTCAG-3' |
|  | *COL1A1* | 5'-CCCCAGCCACAAAGAGTCTA-3’ | 5'-CTGTACGCAGGTGATTGGTG-3' |
